# Supplementary material for: Physiological synaptic activity and recognition memory require astroglial glutamine
Source: Nat Commun. 2022 Feb 8;13:753. doi: 10.1038/s41467-022-28331-7 (PMC8826940; doi:10.1038/s41467-022-28331-7)
Supplement: Supplementary file 1 — Supplementary Information [file 41467_2022_28331_MOESM1_ESM.pdf]

Supplementary Information for

Physiological synaptic activity and recognition memory require astroglial glutamine

Giselle Cheung[1,8], Danijela Bataveljic[1,9‡], Josien Visser[1,2‡], Naresh Kumar[3‡], Julien Moulard[1,2], Glenn Dallérac[1,10], Daria Mozheiko[1,2], Astrid Rollenhagen[4,5], Pascal Ezan[1], Cédric Mongin[3], Oana Chever[1,11], Alexis-Pierre Bemelmans[6], Joachim Lübke[4,5,7], Isabelle Leray[3], Nathalie Rouach[1*]

[*]Correspondence to: nathalie.rouach@college-de-france.fr

This PDF file includes:

Supplementary Figures 1-5

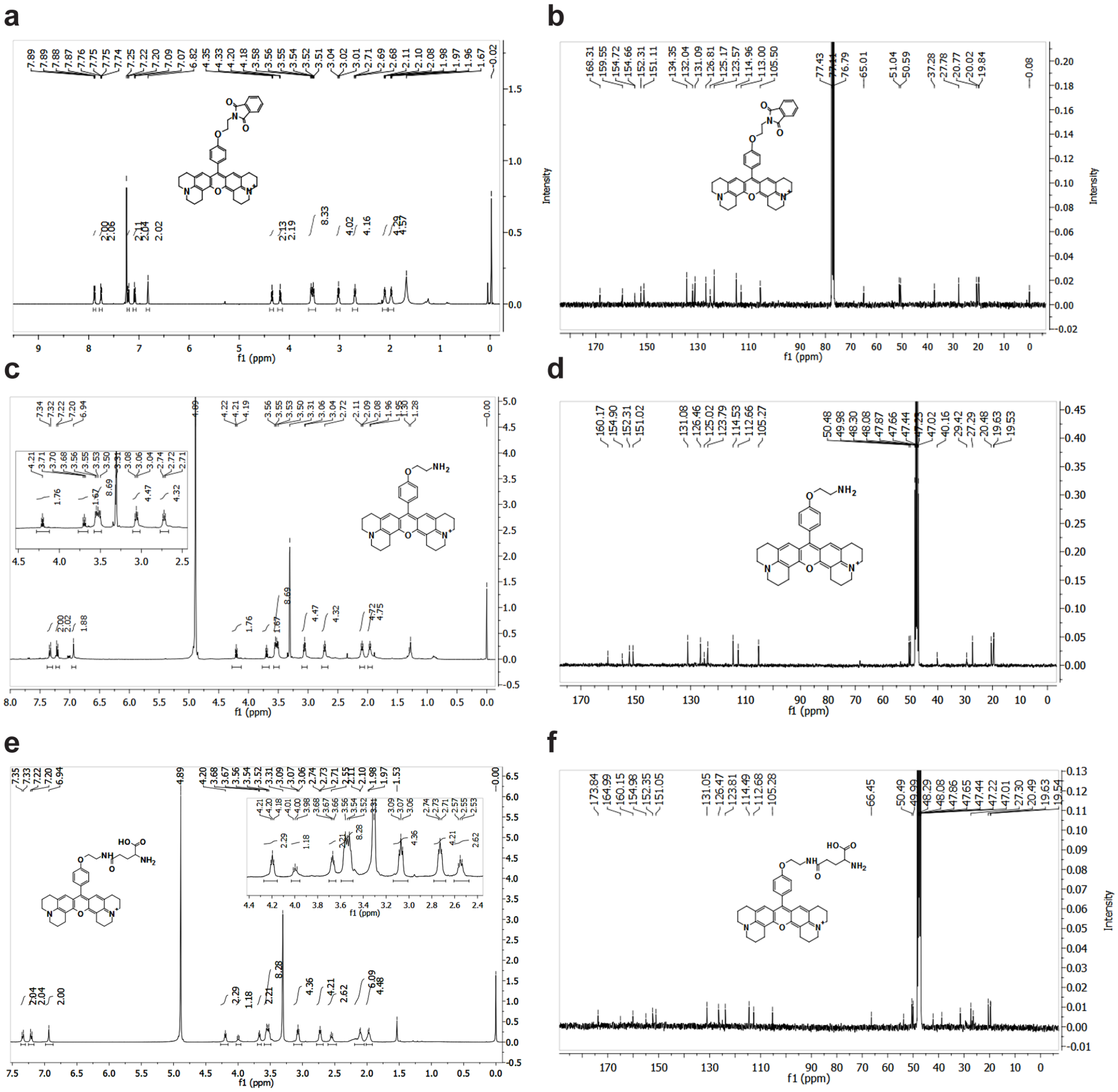

Supplementary Fig. 1. Structural confirmation of intermediate compounds 3, 4, and RhGln.

a, ^{1}H NMR ($CDCl_3$, 400 MHz, 298 K and **b,** ^{13}C NMR ($CDCl_3$, 100 MHz, 298 K)) spectrum of compound **3** is shown. **c,** ^{1}H NMR (CD_3OD, 400 MHz, 298 K) spectrum of compound **4** is shown. Inset displays a zoom on the 4.5–2.5ppm region and **d,** ^{13}C NMR (CD_3OD, 100 MHz, 298 K) spectrum of compound **4**. **e,** ^{1}H NMR (MeOD, 400MHz, 298 K) spectrum of RhGln is shown. Inset displays a zoom on the 4.4–2.4ppm region and **f,** ^{13}C NMR (MeOD, 100 MHz, 298 K) spectrum of RhGln.

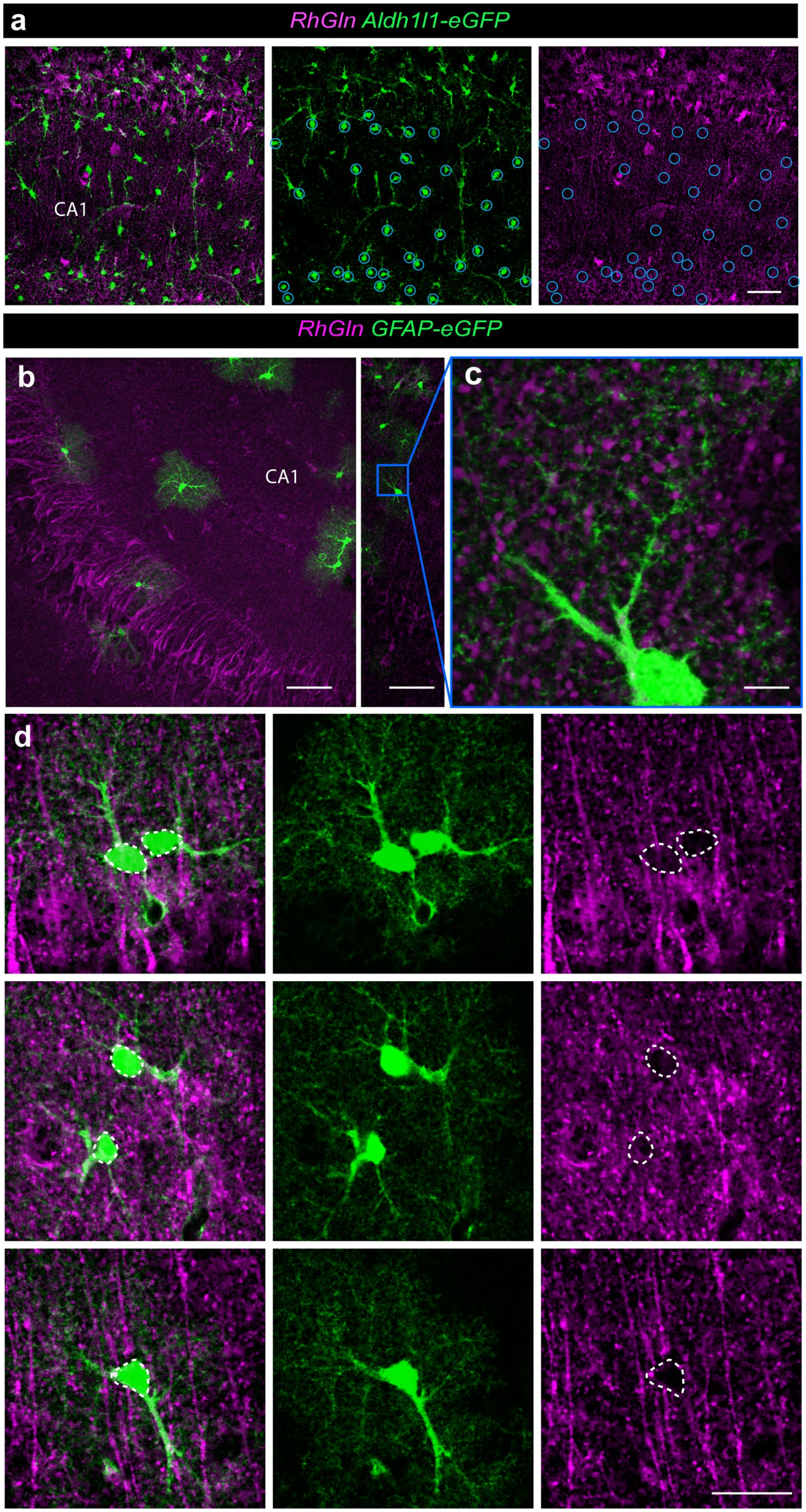

26

Supplementary Fig. 2. Extracellular bulk loaded RhGln does not enter astrocytes. Acute hippocampal slices obtained from either **(a)** Aldh1l1-eGFP or **(b-d)** GFAP-eGFP mice (green) were loaded extracellularly with RhGln (0.05mM, 20min, magenta). **a,** Blue circles mark the absence of RhGln in all Aldh1l1-positive astrocytes. Loading is primarily observed in pyramidal neurons. **b,** Low magnification of CA1 region of GFAP-eGFP slice loaded with RhGln and **c,** high magnification of region marked by blue square in **b** containing astroglial processes **d,** Example images showing that RhGln labeling does not co-localize with GFAP-eGFP expressing astrocytes. Scale bar: **a and b,** 50µm; **c,** 5µm; **d**, 20 µm. Representative images **a - d** are from n=5 replicates.

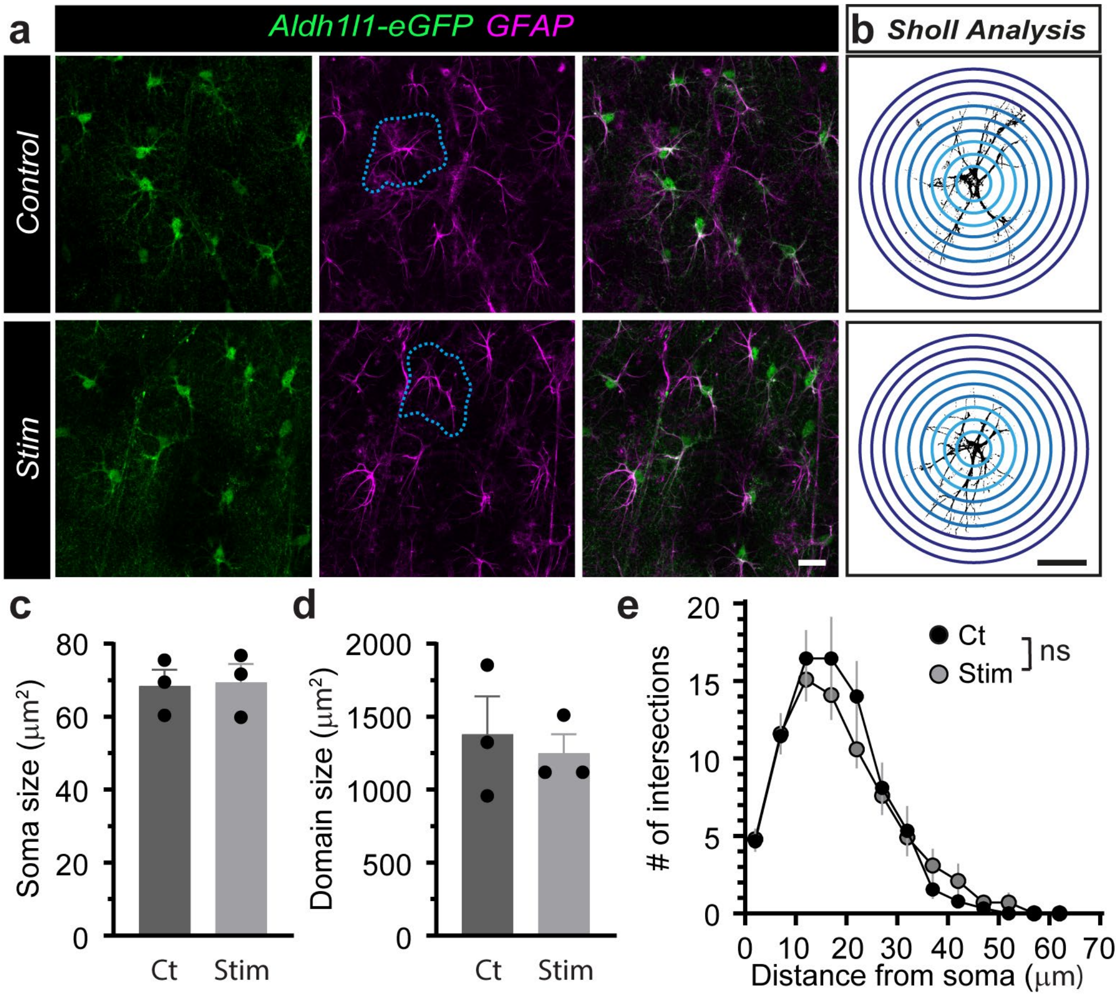

Supplementary Fig. 3. Morphology of astrocytes is unchanged upon stimulation of hippocampal slices. a, Representative images are shown for acute hippocampal slices obtained from Aldh1l1-eGFP mice with or without stimulation of the Schaffer collaterals (10Hz 30s every 3min for 20 min). Aldh1l1-eGFP expression is shown in green and immunostaining for GFAP is shown in magenta. **b,** Sholl analysis was performed as illustrated, where the number of intersections is measured from thresholded GFAP intensity over concentric circles drawn from the cell soma. **c-d**, Soma and domain sizes were measured and quantified from Aldh1l1-eGFP positive somas and GFAP immunostaining, respectively (n=60 cells from 3 independent experiments, $p>0.999$, two-tailed Mann-Whitney test). Domain size was determined by manually drawing an area encompassed by an entire astrocyte (blue dotted line in **a**). Quantification of Sholl analysis in

e showed no significant stimulation-induced difference in astroglial morphology (Ct, n= 9; Stim, n=10, p=0.7129, Two-way ANOVA, ns = not significant). Mean ± SEM in **c** - **e**. Scale bar: **a and b**, 20μm. Source data are provided as a Source Data file.

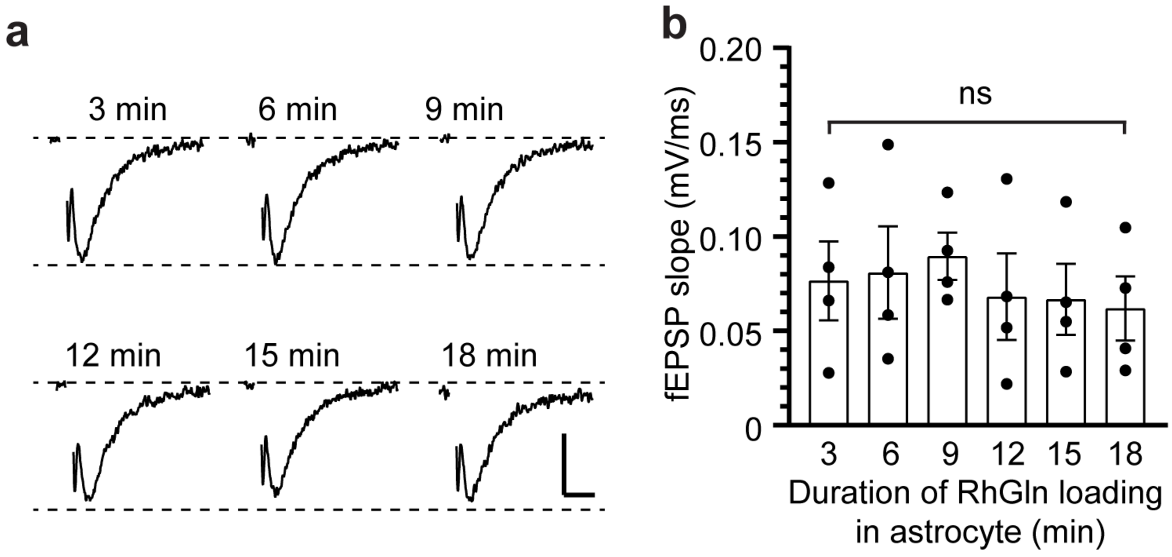

Supplementary Fig. 4. Hippocampal synaptic transmission remains stable during RhGln dye loading in astrocytes. a) Example traces of evoked field excitatory postsynaptic potential (fEPSP) in response to 10Hz stimulation are shown at regular time intervals during dialysis of a single CA1 astrocyte with the RhGln dye (0.8mM). Average traces of the first 300ms of the response to the stimulation are shown. **b)** Quantification of fEPSP slope over time is plotted, showing no significant change, thus indicating a stable hippocampal synaptic transmission during RhGln dye loading in astrocytes (n=4, p=0.9235, One-way ANOVA, , ns = not significant, Mean ± SEM). Scale bar: **a,** 0.2mV, 10ms. Source data are provided as a Source Data file.

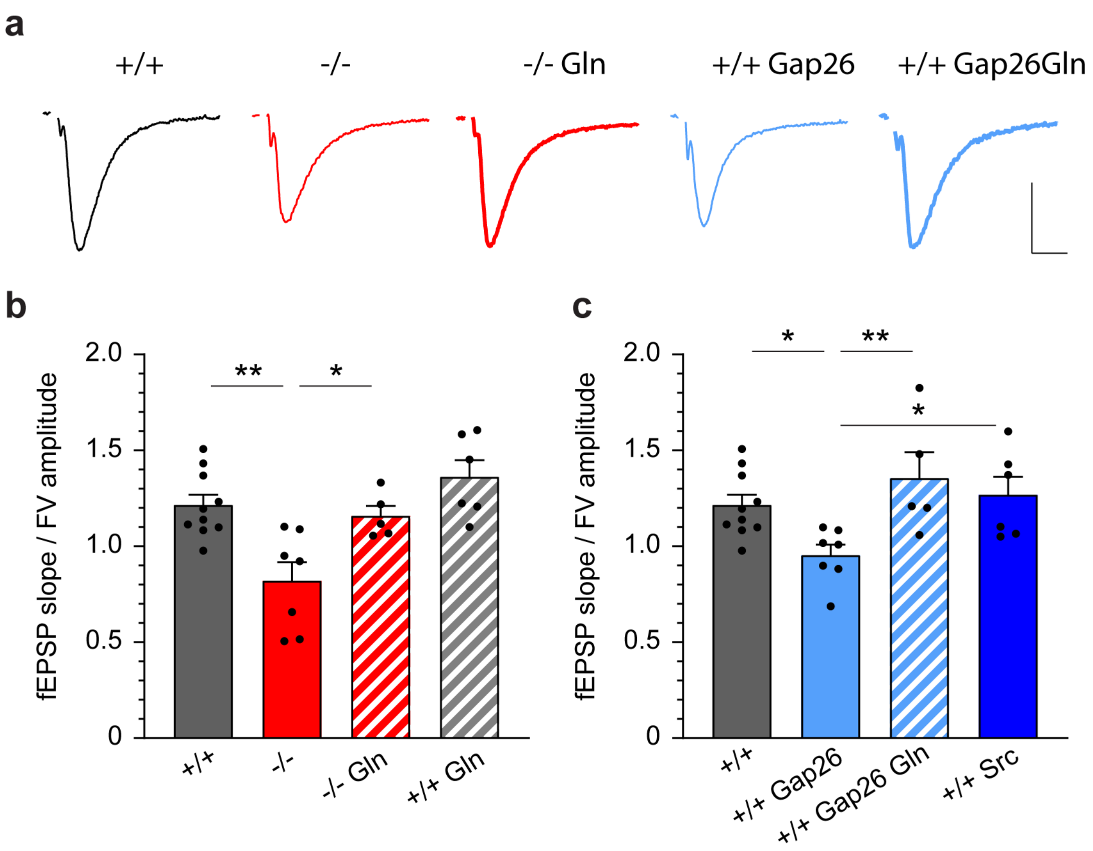

Supplementary Fig. 5. Hippocampal basal synaptic transmission depends on glutamine supply via astroglial Cx43 HC. a, Representative traces showing fEPSPs upon Schaffer collateral single stimulation (10-20μA, 0.1ms). **b,** Quantification of fEPSP slope normalized to fiber volley (FV) amplitude, showing an impairment in synaptic transmission in Cx43-/- slices (-/-, n=7, p=0.0013 with +/+, n=10), which is rescued by exogenous application of glutamine (4mM, -/- Gln, n=5, p=0.0258 with -/-). Glutamine alone did not alter transmission (+/+ Gln, n=6, p=0.5376 with +/+). **c,** Similarly, this effect was mimicked by Gap26 in +/+ slices (+/+ Gap26, n=7, p=0.0458 with +/+, n=10; p=0.0293 with +/+ Src, n=6) and rescued by exogenous glutamine (+/+ Gap26 Gln, n=5, p=0.0065 with +/+ Gap26). All p-values were determined using One-way ANOVA with Bonferroni's post hoc test. Mean ± SEM in **b** - **c**. Scale bar: **a**, 0.2mM, 10ms. Source data are provided as a Source Data file.
