# Supplementary material for: Physiological synaptic activity and recognition memory require astroglial glutamine
Source: Nat Commun. 2022 Feb 8;13:753. doi: 10.1038/s41467-022-28331-7 (PMC8826940; doi:10.1038/s41467-022-28331-7)
Supplement: Supplementary file 3 — Reporting Summary [file 41467_2022_28331_MOESM3_ESM.pdf]

nature research

Corresponding author(s): Nathalie ROUACH

Last updated by author(s): Dec 3, 2021

Reporting Summary

Nature Research wishes to improve the reproducibility of the work that we publish. This form provides structure for consistency and transparency in reporting. For further information on Nature Research policies, see our Editorial Policies and the Editorial Policy Checklist.

Statistics

For all statistical analyses, confirm that the following items are present in the figure legend, table legend, main text, or Methods section.

n/a	Confirmed	
☐	☒	The exact sample size (*n*) for each experimental group/condition, given as a discrete number and unit of measurement
☐	☒	A statement on whether measurements were taken from distinct samples or whether the same sample was measured repeatedly
☐	☒	The statistical test(s) used AND whether they are one- or two-sided<br>*Only common tests should be described solely by name; describe more complex techniques in the Methods section.*
☐	☒	A description of all covariates tested
☐	☒	A description of any assumptions or corrections, such as tests of normality and adjustment for multiple comparisons
☐	☒	A full description of the statistical parameters including central tendency (e.g. means) or other basic estimates (e.g. regression coefficient) AND variation (e.g. standard deviation) or associated estimates of uncertainty (e.g. confidence intervals)
☐	☒	For null hypothesis testing, the test statistic (e.g. *F*, *t*, *r*) with confidence intervals, effect sizes, degrees of freedom and *P* value noted<br>*Give P values as exact values whenever suitable.*
☒	☐	For Bayesian analysis, information on the choice of priors and Markov chain Monte Carlo settings
☒	☐	For hierarchical and complex designs, identification of the appropriate level for tests and full reporting of outcomes
☒	☐	Estimates of effect sizes (e.g. Cohen's *d*, Pearson's *r*), indicating how they were calculated

Our web collection on statistics for biologists contains articles on many of the points above.

Software and code

Policy information about availability of computer code

Data collection	Electrophysiological data acquisition - pClamp 9 (Molecular Devices)<br>Western blot capture software - ImageQuant LAS 4000 (Fujifilm)<br>STED image acquisition - Imspector 16.4 (Abberior)<br>Confocal image acquisition-LAS X (Leica)<br>NOR studies recording - LifeCam Studio software (Microsoft)<br>Time resolved fluorescence - Globals (Laboratory of Fluorescence Dynamics)<br>Electronmicroscopy - Zeiss Libra 120 (Carl Zeiss)
Data analysis	Electrophysiological data analysis - Clampfit 10 (Molecular Devices)<br>Data analysis - Microsoft Excel (Microsoft)<br>Time resolved fluorescence - Globals (Laboratory of Fluorescence Dynamics)<br>NMR - Delta v5.1.3 (Jeol)<br>Electronmicroscopic analysis - SIS Multi Images Acquisition software (Olympus)<br>Statistical analysis - GraphPad Prism v7 and v8 (GraphPad Software, USA)<br>Image analysis - ImageJ/Fiji v1.52j (National Institutes of Health, USA)<br>Image processing - Photoshop CC (Adobe)<br>Image illustration - Illustrator CC (Adobe)<br>Image deconvolution - Huygens (Scientific Volume Imaging B.V.)

For manuscripts utilizing custom algorithms or software that are central to the research but not yet described in published literature, software must be made available to editors and reviewers. We strongly encourage code deposition in a community repository (e.g. GitHub). See the Nature Research guidelines for submitting code & software for further information.

Data

Policy information about availability of data

All manuscripts must include a data availability statement. This statement should provide the following information, where applicable:

- Accession codes, unique identifiers, or web links for publicly available datasets
- A list of figures that have associated raw data
- A description of any restrictions on data availability

We confirm that all relevant data are included in the paper and/or its supplementary information files. Source data are provided with this paper.

Field-specific reporting

Please select the one below that is the best fit for your research. If you are not sure, read the appropriate sections before making your selection.

☒ Life sciences ☐ Behavioural & social sciences ☐ Ecological, evolutionary & environmental sciences

For a reference copy of the document with all sections, see nature.com/documents/nr-reporting-summary-flat.pdf

Life sciences study design

All studies must disclose on these points even when the disclosure is negative.

Sample size	No statistical test was employed to predetermine sample size. Sample size was chosen based on previous literature of similar experiments.<br><br>Rouach, N., Koulakoff, A., Abudara, V., Willecke, K. & Giaume, C. Astroglial metabolic networks sustain hippocampal synaptic transmission. Science 322, 1551-1555, doi:10.1126/science.1164022 (2008).<br>Pannasch, U. et al. Connexin 30 sets synaptic strength by controlling astroglial synapse invasion. Nat Neurosci 17, 549-558, doi:10.1038/nn.3662 (2014).<br>Chever, O., Lee, C. Y. & Rouach, N. Astroglial connexin43 hemichannels tune basal excitatory synaptic transmission. J Neurosci 34, 11228-11232, doi:10.1523/jneurosci.0015-14.2014 (2014).
Data exclusions	There was no data exclusion
Replication	Replications were carried out successfully. A minimum of 3 animals were used as biological replicates. Independent experiments were performed on individual brain slices or tissue sections obtained from different mice. Behavioral experiments were performed with 10 animals in each experimental groups. Details of exact number of replicates carried out for each set of experiment is listed in our figure legends.
Randomization	Mice were selected randomly with correct age and genotype. Hippocampal slices were selected randomly from after acute slice preparation or tissue sectioning. Only male mice were used for behavioral studies which were also randomly selected. For analysis, all images were treated equally in a single set of analysis. No randomization was required.
Blinding	Blinding analysis was performed for EM experiments where image analysis were carried out using coded images and experimenters were unaware of the associated experimental conditions. Blinding was not possible for other experiments as the experimental conditions were always evident to the experimenters.

Reporting for specific materials, systems and methods

We require information from authors about some types of materials, experimental systems and methods used in many studies. Here, indicate whether each material, system or method listed is relevant to your study. If you are not sure if a list item applies to your research, read the appropriate section before selecting a response.

Materials & experimental systems

n/a	Involved in the study	
☐	☒	Antibodies
☐	☒	Eukaryotic cell lines
☒	☐	Palaeontology and archaeology
☐	☒	Animals and other organisms
☒	☐	Human research participants
☒	☐	Clinical data
☒	☐	Dual use research of concern

Methods

n/a	Involved in the study	
☒	☐	ChIP-seq
☒	☐	Flow cytometry
☒	☐	MRI-based neuroimaging

Antibodies

Antibodies used	Polyclonal chicken anti-GFP (1:500, AB13970, Abcam)<br>monoclonal mouse anti-VGlut1 (1:200, 135511, clone 317G6, Synaptic Systems)<br>monoclonal mouse anti-Cx43 (1:500, 610-062, clone 2, BD Biosciences)<br>polyclonal rabbit anti-Cx43 (1:500, 71-2200, Zymed Laboratories)<br>monoclonal mouse anti-Cx43 (1:250, C8093, CXN-6, Chemicon)<br>monoclonal mouse anti-GFAP (1:500, G3893, G-A-5, Sigma)<br>monoclonal mouse anti-GAPDH-peroxidase (1:1000, G9295, GFAPDH-71.1, Sigma)<br><br>goat anti-mouse IgG conjugated to Alexa 488, 555 or 647 (1:2000; A11029, A21424 or A21235, Life Technologies)<br>goat anti-rabbit IgG conjugated to Alexa 488 or 555 (1:2000, A11034 or A21429, Life Technologies)<br>goat anti-chicken IgG conjugated to Alexa 488 (1:2000, A11039, Life Technologies)<br>goat anti-mouse IgG-HRP (1:2500, sc-2005, Santa-Cruz)<br>goat anti-rabbit IgG-HRP (1:2500, sc-2004, Santa-Cruz)<br>gold-conjugated anti-mouse secondary antibody (1:40, British Biocell)
Validation	All antibodies used in this study are commercially available and was validated by literature and manufacturer. They were used according to recommendations by manufacturers.<br><br>Polyclonal chicken anti-GFP antibody (AB13970, Abcam) has been validated for use in western blotting and immunocytochemistry/ immunofluroescence as described on product website.<br>monoclonal mouse anti-VGlut1 (135511, Synaptic Systems) has been validated to be reactive to mouse and for use in western blotting and immunocytochemistry/immunofluroescence as described on product website.<br>monoclonal mouse anti-Cx43 (610-062, BD Biosciences) has been validated to be reactive to mouse and for use in western blotting and immunocytochemistry/immunofluroescence as described on product website.<br>polyclonal rabbit anti-Cx43 (71-2200, Zymed Laboratories) has been validated to be reactive to mouse and for use in western blotting and immunocytochemistry/immunofluroescence as described on product website.<br>monoclonal mouse anti-Cx43 (C8093, Chemicon) has been validated to be reactive to mouse and for use in western blotting and immunocytochemistry/immunofluroescence as described on product website.<br>monoclonal mouse anti-GFAP (G3893, Sigma) has been validated to be reactive to rat and for use in immunocytochemistry/ immunofluroescence as described on product website. The use of this antibodies in mice have been extensively documented as shown by citation on product website.<br>monoclonal mouse anti-GAPDH-peroxidase (G9295, Sigma) has been validated to be reactive to mouse and for use in western blotting as described on product website.

Eukaryotic cell lines

Policy information about cell lines

Cell line source(s)	HEK293T cells (human, ATCC CRL-1573)
Authentication	None of the cell lines have been authenticated.
Mycoplasma contamination	Absence of mycoplasma contamination of the cell line was assessed every 6 months by pcr methods
Commonly misidentified lines<br>(See ICLAC register)	No commonly misidentified cell lines were used.

Animals and other organisms

Policy information about studies involving animals; ARRIVE guidelines recommended for reporting animal research

Laboratory animals	Wild-type C57BL/6j mice were used at the age between postnatal days 17-30 or 3 months old. GFAP-eGFP mice of C57BL/6j background were used at the age between postnatal days 17-30. Aldh1l1-eGFP mice of FVB/N background were used at the age between postnatal days 17-30. Cx43fl/fl:hGFAP-Cre mice of C57BL/6j background were used at the age between postnatal days 17-30. Kir4.1 fl/fl:hGFAP-Cre mice of C57BL/6j background were used at the age between postnatal days 17-30. Both male and female mice were used.
Wild animals	No wild animals were used in the study.
Field-collected samples	No field collected samples were used in the study.
Ethics oversight	All procedures on animals were performed according to the guidelines of European Community Council Directives of 01/01/2013 (2010/63/EU) and our local animal care committee (Center for Interdisciplinary Research in Biology in College de France).

Note that full information on the approval of the study protocol must also be provided in the manuscript.
